# Variation in the prion protein gene (*PRNP*) sequence of wild deer in Great Britain and mainland Europe

**DOI:** 10.1186/s13567-019-0675-6

**Published:** 2019-07-31

**Authors:** Amy L. Robinson, Helen Williamson, Mariella E. Güere, Helene Tharaldsen, Karis Baker, Stephanie L. Smith, Sílvia Pérez-Espona, Jarmila Krojerová-Prokešová, Josephine M. Pemberton, Wilfred Goldmann, Fiona Houston

**Affiliations:** 1 Division of Infection and Immunity, The Roslin Institute and The Royal Dick School of Veterinary Studies, University of Edinburgh, Midlothian, EH259RG UK; 2 Norwegian University of Life Sciences, Faculty of Veterinary Medicine, Oslo, Norway; 3 Department of Biosciences, Durham University, South Road, Durham, DH1 3LE UK; 4 The Royal Dick School of Veterinary Studies, University of Edinburgh, Midlothian, EH259RG UK; 5 Institute of Vertebrate Biology of the Czech Academy of Sciences, Květná 8, 603 65 Brno, Czech Republic; 6 Department of Zoology, Fisheries, Hydrobiology and Apiculture, Faculty of AgriSciences, Mendel University in Brno, Zemědělská 1, 613 00 Brno, Czech Republic; 7 Institute of Evolutionary Biology, School of Biological Sciences, University of Edinburgh, Edinburgh, UK

## Abstract

Susceptibility to prion diseases is largely determined by the sequence of the prion protein gene (*PRNP*), which encodes the prion protein (PrP). The recent emergence of chronic wasting disease (CWD) in Europe has highlighted the need to investigate *PRNP* gene diversity in European deer species, to better predict their susceptibility to CWD. Here we report a large genetic survey of six British deer species, including red (*Cervus elaphus*), sika (*Cervus nippon*), roe (*Capreolus capreolus*), fallow (*Dama dama*), muntjac (*Muntiacus reevesii*), and Chinese water deer (*Hydropotes inermis*), which establishes *PRNP* haplotype and genotype frequencies. Two smaller data sets from red deer in Norway and the Czech Republic are also included for comparison. Overall red deer show the most *PRNP* variation, with non-synonymous/coding polymorphisms at codons 98, 168, 226 and 247, which vary markedly in frequency between different regions. Polymorphisms P168S and I247L were only found in Scottish and Czech populations, respectively. T98A was found in all populations except Norway and the south of England. Significant regional differences in genotype frequencies were observed within both British and European red deer populations. Other deer species showed less variation, particularly roe and fallow deer, in which identical *PRNP* gene sequences were found in all individuals analysed. Based on comparison with *PRNP* sequences of North American cervids affected by CWD and limited experimental challenge data, these results suggest that a high proportion of wild deer in Great Britain may be susceptible to CWD.

## Introduction

Chronic wasting disease (CWD) is a prion disease of cervid species that is widespread in North America and has recently emerged in Europe. It belongs to a family of diseases termed transmissible spongiform encephalopathies (TSEs), which cause progressive and invariably fatal neurodegenerative disorders in humans and animals. CWD was first described in the 1960s as a wasting syndrome of captive mule deer (*Odocoileus hemionus hemionus*) and black-tailed deer (*Odocoileus hemionus columbianus*) in Colorado wildlife facilities [1]. It has since been identified in North America in wapiti (elk, *Cervus canadensis*) white-tailed deer (*Odocoileus virginianus*), moose (*Alces alces*) and a single captive red deer (*Cervus elaphus*) [2, 3]. It is currently the only animal prion disease recognised in both captive and wild populations, and efficient transmission has led to spread of the disease across North America. At the time of writing, CWD is present in at least 25 US states and three Canadian provinces [4], as well in South Korea, introduced via importation of infected wapiti [5].

Member States of the European Union (EU) and Norway were believed to be free of CWD based on a survey conducted between 2006 and 2009 on 3274 farmed and 10 049 wild deer [6], with the vast majority being red deer (10 110). However, the first European case of CWD, and the first natural case in a reindeer (*Rangifer tarandus*) was identified in Norway in March 2016 [7]. Subsequent culling of the affected reindeer population resulted in detection of another 18 infected animals, and increased surveillance in Scandinavia has identified further “atypical” cases in three moose and a single red deer in separated locations in Norway, and individual moose in Finland and Sweden. Although the origins of CWD in Scandinavia are uncertain, the wide geographic distribution of cases raises concern that disease may have been present and undetected for some time [8–11].

The development of prion diseases is associated with misfolding of the prion protein (PrP^C^) into a protease-resistant form (PrP^Sc^). The sequence of the open reading frame (ORF) of the gene encoding the prion protein (*PRNP)* is strongly associated with susceptibility to prion diseases. This association has been exploited in breeding programmes such as the National Scrapie Plan in the UK, which selectively bred for disease resistant *PRNP* genotypes to reduce the incidence of classical scrapie in sheep [12, 13]. Cervid *PRNP* genetics have been widely studied in North America, where both natural and experimental infection of deer species has allowed for the identification of *PRNP* polymorphisms that are associated with reduced incidence of disease and/or slower disease progression. The first identified was M132L in wapiti, which encodes a methionine to leucine change at codon 132. Genotype surveys of CWD affected deer populations have produced conflicting evidence regarding the association of codon 132 variation with incidence of disease [14, 15], but CWD challenges of deer [16, 17] and transgenic mice [18] suggest that the 132L variant reduces susceptibility to infection. Other important *PRNP* polymorphisms associated with CWD susceptibility include S225F in mule deer, [19–22], Q95H, G96S and A116G in white-tailed deer [23–28]. Although many of these *PRNP* polymorphisms have been associated with reduced susceptibility to CWD, none of them appear to confer complete resistance.

Experimental transmissions of CWD provide some evidence of susceptibility in deer species found in Europe. CWD has been transmitted orally to red deer [29], reindeer [30] and Reeves’ muntjac deer [31, 32]. Fallow deer have been infected via intracerebral inoculation [33], but failed to become infected when co-grazed with infected mule deer [34]. However, due to small sample sizes in all these studies, it is difficult to draw associations between susceptibility to experimental CWD infection and host *PRNP* genotype. It is also not known if the *PRNP* genotypes of the experimental animals used in these North American studies are representative of the genetics of native wild European deer populations.

The extent of *PRNP* sequence variation in European deer species has not previously been studied in detail, and this genetic information will be critically important in estimating potential susceptibility of these populations to emerging CWD and informing risk assessment and control/surveillance strategies. Novel *PRNP* variants (not seen in North American cervids) that show evidence of association with reduced susceptibility or resistance to CWD have the potential to be used in breeding programmes for captive and farmed deer. The primary aims of this study were (i) to perform a comprehensive survey of the protein-coding sequence of the *PRNP* gene in the six major free-ranging or wild species of deer found in Great Britain (GB) and (ii) to compare the *PRNP* genotype distributions in British and European populations of red deer, as red deer are one of the most numerous wild species in both GB and mainland Europe, and are also an economically important game species due to revenue generated via hunting, tourism and venison production [35].

## Materials and methods

### Samples from British deer species

**Figure 1:**
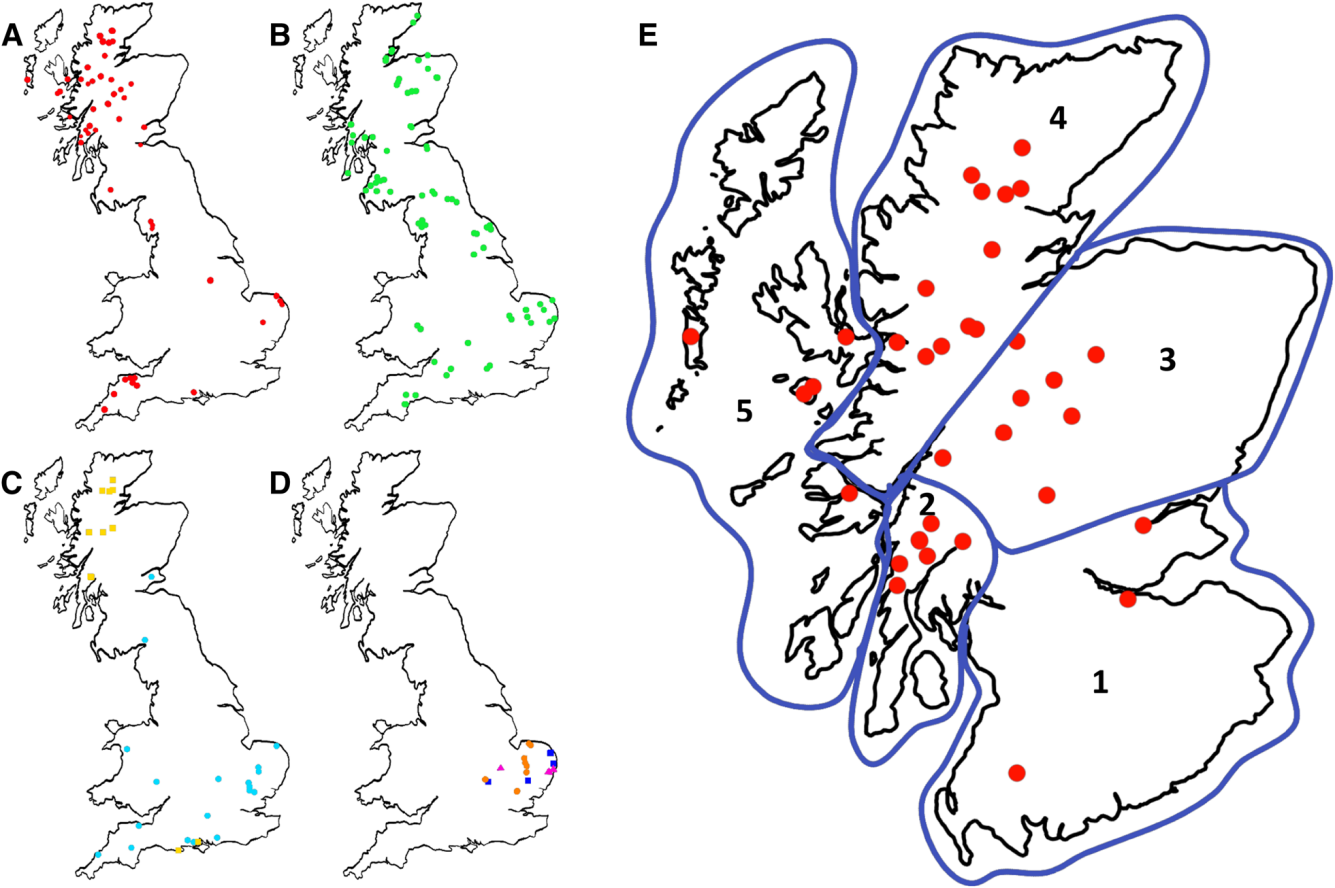
**Maps to show the sampling locations of deer samples from Great Britain.** Each point represents one sampling location with ≤ 28 animals sampled. **A** Red deer (red circles), **B** roe deer (green circles), **C** sika (yellow squares) and fallow (blue circles), **D** muntjac (orange circles) and Chinese water deer (dark blue squares), or both muntjac and Chinese water deer (pink triangles). **E** Red deer samples in Scotland separated into regions: South Scotland (1), Argyll (2), Central Scotland (3), North Highlands (4) and Hebrides (5)

A total of 1003 samples collected from wild deer in GB (England, Scotland and Wales) were analysed. Archived DNA samples were provided by JP, SPE and SLS for red deer, sika deer, and red/sika hybrids, and by KB for roe deer. The procedures for sample collection and genomic DNA preparation for these archives have been described previously [36–38]. Additional samples were collected by stalkers in the British Deer Society to give representation of geographical areas and species not covered by the existing archives. These included samples collected during routine culling, and samples collected from culled deer at a meat processing facility. Samples consisted of small sections of ear tips, which were preserved in 20% DMSO/saturated NaCl solution during transportation/storage [39] For each sample, further information including the sex, estimated age, and postcode/grid coordinates of the location of the animal, were recorded. The distribution of sampling sites for each deer species are shown in Figure 1.

In total, samples from 480 red deer were analysed, of which 388 were from the archived collection, and 92 samples were collected by BDS deer stalkers. Samples from 83 sika deer were analysed, 73 samples were archived DNA, and 10 stalker collected samples from the South of England. All samples collected by members of the BDS were identified by animal phenotype only, whereas archived DNA samples of red and sika deer were selected both on phenotypical appearance and microsatellite genotyping data [38, 40], whereby a Q value of Q = 1 indicated a pure red deer, Q = 0 indicated a pure sika deer. Nine sika-red hybrids were also included in this study and were classified as having 0.091 ≤ Q ≤ 0.772.

For analysis red deer populations were separated into the following regions: Southern England (mainly Exmoor National Park) (*n* = 78), Northern England (Lake District) (*n* = 32), Southern Scotland (*n* = 23), Argyll (*n* = 47), Central Highlands (*n* = 142), Northern Highlands (*n* = 114) and Hebrides (*n* = 44) (Figure 1).

A total of 297 samples from roe deer were analysed, 171 from a DNA archive [37], and 126 collected by BDS deer stalkers. Samples from 66 fallow deer and 41 muntjac deer were collected by BDS deer stalkers. A total of 27 Chinese Water deer samples were analysed, 3 from a DNA archive [41] and 24 supplied by BDS deer stalkers.

### Samples from other European red deer

Genotype analysis was also performed on 50 wild red deer collected from six counties in Norway, and 66 archived DNA samples from wild red deer (*n* = 46) and wild sika (*n* = 20) collected in five regions of the Czech Republic [42, 43]. In central Europe, 2 distinct lineages of red deer are present. Of the 46 Czech red deer, 24 were of Western lineage, and 22 of Eastern lineage.

### Extraction and purification of genomic DNA

Genomic DNA was extracted from small sections of ear-tip tissue, digested with proteinase K (PK) at 37 °C overnight, followed by standard phenol/chloroform purification, ethanol precipitation and re-suspension in 1XTE buffer, as described previously [44]. Norwegian samples were prepared from brain tissue using DNeasy^®^ Blood & Tissue Kit (Qiagen, Oslo, Norway) according to manufacturer’s instructions.

### PRNP gene amplification and sequencing: British and Czech samples

The ORF of cervid *PRNP* (771 bp) was amplified by PCR using AmpliTaq Gold 360 master mix (Thermo Fisher Scientific) and primer pairs − 143d (ATGGAATGTGAAGAACATTTATGACCTA) or − 213d (AGGTCAACTTTGTCCTTGGAGGAG) with + 139u (TAAGCGCCAAGGGTATTAGCAT) or AR2 (GCAAGAAATGAGACACCACCAC) for British and Czech red, roe and sika deer, − 213d and AR3 (ACCACTACAGGGCTGCAGGTA) for fallow and Chinese water deer, and − 213d and AR2 (GCAAGAAATGAGACACCACCAC) for muntjac deer. PCR conditions were 95 °C for 5 min followed by 40 cycles of 95 °C for 30 s, 61 °C for 30 s and 72 °C for 1 min, and a final 72 °C for 10 min. PCR fragments were sequenced by sanger sequencing using primer + 70u (GCTGCAGGTAGATACTCCCTC) and BigDye^®^ reagents (Life Technologies, Paisley, UK) as recommended by the manufacturer. Sequence data were analysed using Chromas and DNAStar Lasergene version 14.

### PRNP gene amplification and sequencing: Norwegian samples

The ORF was amplified using PCR primers Ce19_F (ATTTTGCAGATAAGTCATC) and Ce 778_R (AGAAGATAATGAAAACAGGAAG) [14]. PCR conditions were 95 °C for 2 min, followed by 36 cycles at 95 °C for 30 s, 51 °C for 30 s and 72 °C for 45 s, then a final cycle at 72 °C for 10 min. PCR fragments were directly sequenced using Ce19_F and Ce 778_R and BigDye^®^ reagents (Life Technologies, Paisley, UK) as recommended by the manufacturer. Sequence data were analysed using Seqscape v3.0 software, Sequence Scanner v2.0 (Applied Biosystems) and MEGA7 version 7.0.26 [45].

### Statistical analysis

The Chi square test was used to compare haplotype frequencies between different regions and countries.

### Accession numbers

Sequences were deposited in Genbank with the following accession numbers: *Capreolus capreolus* MK103016; *Dama dama* MK103017; *Cervus nippon* MK103018, MK103019; *Muntiacus reeveesi* MK103020–MK103023; *Hydropotes inermis* MK103024–MK103026; *Cervus elaphus* with 247L MK103027.

## Results

The ORF encoded by exon 3 of the *PRNP* gene is highly conserved among cervid species, and non-synonymous polymorphisms identified in individual species are usually reported as variations from a consensus sequence [25]. Following this convention, the amino acid substitutions resulting from *PRNP* sequence polymorphisms identified in the deer species examined in our survey are summarized in Table 1.

**Table 1:** **Amino acid variation within the ORF of cervid** ***PRNP*** **in the surveyed deer species**

	*PRNP* codon number					
	98	100	138	168	226	247
Consensus sequence	T	S	S	P	Q	I
Red *Cervus elaphus*						
TPQ	–	–	–	–	–	–
TPE	–	–	–	–	E	–
APQ	A	–	–	–	–	–
APQ-L_247_	A	–	–	–	–	L
ASQ	A	–	–	S	–	–
Roe *Capreolus capreolus*						
TPQ	–	–	–	–	–	–
Sika *Cervus nippon*						
TPQ	–	–	–	–	–	–
Fallow *Dama dama*						
TPE-N_138_	–	–	N	–	E	–
Muntjac *Muntiacus reevesii*						
SPQ	S	–	–	–	–	–
Chinese water deer						
*Hydropotes intermis*						
TPQ	–	–	–	–	–	–
TPQ-N_100_	–	N	–	–	–	–

### British red deer

Four single nucleotide polymorphisms (SNPs) were identified in 480 British red deer samples: a synonymous SNP at codon 136 (t/c, nucleotide position 408), and three non-synonymous polymorphisms at codons 98 (a/g, nucleotide position 292), 168 (c/t, nucleotide position 502) and 226 (g/c, nucleotide position 676) giving rise to amino acid changes threonine to alanine (T98A), proline to serine (P168S) and glutamine to glutamic acid (Q226E), respectively. Linkage was found between positions 408 and 676 (codons 136 and 226), such that the haplotypes were either 408t-676c or 408c-676g. The SNPs resulting in substitutions at positions 98 and 168 occurred exclusively on a Q226 background and the following four haplotypes were inferred: T_98_-P_168_-E_226_ (TPE), T_98_-P_168_-Q_226_ (TPQ), A_98_-P_168_-Q_226_ (APQ) and A_98_-S_168_-Q_226_ (ASQ).

### Norwegian and Czech red deer

Only the TPE and TPQ haplotypes were identified in Norwegian red deer. The synonymous codon 136 polymorphism was observed in the same haplotype linkage as described above. Czech red deer had TPE, TPQ and APQ haplotypes, as well as a novel non-synonymous SNP at codon 247 (a/c nucleotide position 739) leading to a conservative isoleucine to leucine substitution which occurred in linkage with APQ (APQ-L_247_).

### British sika, Czech sika and sika-red deer hybrids

Scottish pure sika (*n* = 73), Czech sika (*n* = 20), English sika (*n* = 8/10) were all homozygous for the TPQ haplotype. Both British and Czech sika had a synonymous polymorphism at codon 133 (g/c, position 399) at frequencies of 0.08% and 0.1% respectively. Of the nine genetically determined sika-red hybrids from Argyll (0.091 ≤ Q ≤ 0.772) three were homozygous TPQ/TPQ, four were heterozygous TPE/TPQ, and two were heterozygous for the APQ haplotype (APQ/TPQ and APQ/TPE). The two animals heterozygous for the APQ haplotype were characterised as red deer-like (Q = 0.71, 0.74), as were two of four TPE/TPQ heterozygotes (Q = 0.77, 0.62). Of the 10 English animals identified as sika by phenotype only, two were TPE/TPQ heterozygous and were therefore possibly sika-like hybrids.

### Roe and fallow deer

The *PRNP* ORF of British roe deer (*n* = 297) all encoded the haplotype described above as TPQ. Roe deer were monomorphic in *PRNP* sequence, with no variation in nucleotide sequence in any of the animals analysed. Likewise, fallow deer (*n* = 66) showed no variation in *PRNP* sequence and encoded the TPE haplotype with an additional amino acid substitution of asparagine for serine at codon 138 (TPE-N_138_).

### Muntjac and Chinese water deer

The *PRNP* ORF of all 41 muntjac deer analysed encoded serine at position 98, allowing their *PRNP* haplotype to be described as S_98_P_168_Q_226_ (SPQ). In addition, synonymous polymorphisms were identified at nucleotide positions 15 (c/t), 126 (g/a) and 606 (c/t) at varying frequencies. The *PRNP* coding region of 27 Chinese water deer revealed two amino acid sequence polymorphisms; a serine to asparagine substitution at codon 100 (g/a, nucleotide position 299) and a 24 bp deletion of the octapeptide repeat (ccccatggaggtggctggggtcag) which corresponds to the in-frame loss of the amino acid sequence PHGGGWGQ. In most species, a glycine-rich peptide sequence (octapeptide repeat) is present in five consecutive copies in the N terminal region of wild type PrP^C^, whilst Chinese water deer had a four octapeptide repeat haplotype. From the genotypes it was inferred that the deletion was linked to 100S (100S-Δ_71–78_). The haplotype frequencies were 0.21 for 100S, 0.25 for 100SΔ_71–78_, and 0.54 for 100 N. Seven animals were heterozygous for 100SΔ_71–78_, whilst three were homozygous S_100_Δ_71–78_/S_100_Δ_71–78_. The most common genotype in this sampling was N_100_/N_100_ (0.35).

### Red deer genotype and haplotype frequencies and distributions

Red deer showed the greatest variation in *PRNP* sequence amongst the six deer species surveyed, with four haplotypes identified in the British population. Regional variation was evident within Great Britain in the distribution and frequency of the four *PRNP* haplotypes and resulting genotypes (Tables 2 and 3). For the TPQ and TPE haplotypes, frequencies in different regions of Scotland and England ranged from 0.11 to 0.62 and 0.31 to 0.88 respectively. The difference was most striking in Southern England, which had a very high frequency (0.88) of the TPE haplotype, whereas in Northern England, the TPQ haplotype was predominant (0.67). The APQ and ASQ haplotypes were found almost exclusively in Scottish red deer, with only one heterozygous TPE/APQ individual identified in Northern England. The frequencies of APQ and ASQ haplotypes showed regional variation within Scotland, with the highest frequencies seen in the Central Highlands, Argyll and Southern Scotland (Table 3). When compared using the Chi squared test, the Central Highlands, Lowlands and Argyll were significantly different in haplotype frequency when compared to the Northern Highlands and Hebrides (*P* < 0.05), as were the overall haplotype frequencies of England and Scotland (*P* < 0.05).

**Table 2:** **Genotype frequencies of** ***PRNP*** **polymorphisms in British red deer populations**

98	168	226	England			Scotland					
			Total	S	N	Total	SS	A	CH	NH	H
TT	PP	EE	58 (64)	78 (61)	9 (3)	29 (107)	31 (7)	28 (13)	13 (19)	41 (47)	48 (21)
TT	PP	QE	26 (28)	19 (15)	41 (13)	28 (102)	22 (5)	15 (7)	28 (39)	34 (39)	27 (12)
TT	PP	QQ	15 (17)	3 (2)	47 (15)	10 (36)	9 (2)	8 (4)	11 (15)	10 (11)	9 (4)
TA	PP	QE	1 (1)	–	3 (1)	14 (52)	26 (6)	19 (9)	16 (23)	9 (10)	9 (4)
TA	PP	QQ	–	–	–	8 (31)	4 (1)	13 (6)	11 (16)	5 (6)	5 (2)
AA	PP	QQ	–	–	–	3 (13)	–	11 (5)	4 (6)	1 (1)	2 (1)
TA	PS	QE	–	–	–	4 (14)	4 (1)	4 (2)	8 (11)	–	–
TA	PS	QQ	–	–	–	2 (8)	4 (1)	2 (1)	4 (6)	–	–
AA	PS	QQ	–	–	–	2 (7)	–	–	5 (7)	–	–
AA	SS	QQ	–	–	–	–	–	–	–	–	–
			100 (110)	100 (78)	100 (32)	100 (370)	100 (23)	100 (47)	100 (142)	100 (114)	100 (44)

S: Southern England, N: Northern England, SS: Southern Scotland, A: Argyll, CH: Central Highlands, NH: Northern Highlands, H: Hebrides.

Percentage frequencies are given with animal numbers in brackets.

**Table 3:** **Haplotype frequencies of** ***PRNP*** **polymorphisms in British red deer populations**

Haplotype	Southern England	Northern England	Southern Scotland	Argyll	Central Highlands	Northern Highlands	Hebrides	GB average
TPE	0.88	0.31	0.57	0.47	0.39	0.63	0.66	0.56
TPQ	0.12	0.67	0.24	0.23	0.32	0.29	0.25	0.29
APQ	0	0.02	0.15	0.27	0.20	0.08	0.09	0.12
ASQ	0	0	0.04	0.03	0.09	0	0	0.03
Total	1.00	1.00	1.00	1.00	1.00	1.00	1.00	1.00

The haplotype frequencies in GB were also compared by Chi squared test with those in red deer from Norway and the Czech Republic, as well as previously published surveys of Scotland, Italy and Spain [46, 47] (Table 4). The number of samples was too low to assess the extent of regional variation within Norway and the Czech Republic. In Norway, where only the TPQ and TPE haplotypes were identified, haplotype frequencies were very similar to those found in Southern England, i.e. 0.89 for TPE and 0.11 for TPQ. In the Czech Republic, frequencies of the TPQ and TPE haplotypes were approximately equal (0.35 and 0.44, respectively), while the APQ and APQ-L_247_ haplotypes were present at frequencies of 0.16 and 0.05, respectively. The APQ-L_147_ haplotype was only seen in the Czech red deer belonging to the Western lineage (*n* = 24) [43]. The haplotype frequencies presented in Table 4 were compared across countries, and whilst most differences between countries were statistically significant (*P* < 0.05), those in Spain and the Czech Republic were not.

**Table 4:** **Comparison of haplotype frequencies across European red deer populations**

	TPE	TPQ	APQ	ASQ	Total animals	References
England	0.71	0.28	0^a^	0	110	
Scotland	0.52	0.29	0.16	0.04	370	
Scotland	0.69	0.27	0.05	0	132	[46]
Norway	0.89	0.11	0	0	50	
Czech	0.35	0.44	0.21^b^	0	46	
Spain	0.36	0.43	0.21	0	209	[47]
Italy	0.28	0.62	0.10	0	191	[46]

^a^A single animal was identified with the APQ genotype.

^b^The L_247_ polymorphism was present on an APQ background at a frequency of 0.05.

## Discussion

This study represents the largest survey to date of *PRNP* genetic variation in the wild (free-ranging) deer population of any European country and is the first to include free-ranging muntjac and Chinese water deer. The analysis included over 1003 animals from six different species of deer present in Britain, as well as red deer and sika deer from mainland Europe. Ten haplotypes were identified, five of which were present in red deer. The majority of polymorphisms identified in the ORF (exon 3) of the *PRNP* gene have been reported previously [32, 46–49], apart from the I247L polymorphism in red deer from the Czech Republic, and the octapeptide repeat deletion in Chinese water deer. Deletion of the third glycine-rich octapeptide repeat is not an uncommon variation, having previously been described in chamois [47], lions [50], and some primates [51]. This is the first time a functional octapeptide deletion variant has been described in deer.

Red deer showed the greatest variation in *PRNP* sequence, with four haplotypes identified in Great Britain, designated TPQ, TPE, APQ and ASQ. The TPQ, TPE and APQ haplotypes have been previously reported in surveys of Italian, Scottish (as haplotypes 1, 8 and 10) [46] and Spanish red deer [47]. The ASQ haplotype was not identified in Italy and Spain, and only reported previously in a single Scottish red deer [46]. Non-synonymous polymorphisms previously identified in Italian red deer at codons 59 (G59S) and 208 (M208I) in single individuals [46, 52], as well as low frequency synonymous polymorphisms at codons 15, 21, 59, 78 and 79 [46] were not present in our surveyed populations.

Taking our results together with previously published surveys, the total number of European red deer analysed for *PRNP* sequence variation stands at 1108 individuals. In every region or country surveyed, TPQ and TPE haplotypes were present at the highest frequencies (Tables 3 and 4). In comparison to other European red deer populations, there was a high frequency of the TPE haplotype in South England (predominantly the Exmoor National Park) and in Norway, which is possibly due to historical population declines of red deer with refugial populations in these areas [53]. The APQ haplotype was absent from both Southern England and Norway but was found at similar frequencies in Scottish (0.16), Czech (0.16), Spanish (0.21) and Italian (0.10) red deer [46, 47]. In our study, it was evident that the frequency of the APQ haplotype showed marked variation between different Scottish regions (Table 3). This may explain why our frequency of the APQ haplotype was higher (0.16) than the previous Scottish survey (0.05); in the previous study, samples were collected from the Isle of Rum and an unspecified location on mainland Scotland, which may not have coincided with regions where we identified the highest APQ haplotype frequencies. The ASQ and APQ-L_247_ haplotypes were present at frequencies of 0.04 and 0.05 in Scottish and Czech red deer populations respectively and may be unique to these populations. Our results emphasize that there are large regional variations in *PRNP* genotype frequencies in British red deer populations, and similar variation in genotype frequencies between the red deer populations surveyed in European countries to date. Regional variations in the genetics of Scottish red deer populations have been previously reported, in part due to natural barriers [36] and in part due to introductions of non-native red deer into Scotland from England and further afield [54].

In contrast to red deer, our roe deer samples showed no variation in their *PRNP* sequence. Whilst the number of analysed animals across studies stands now at 541, only one synonymous change at codon 24 has previously been reported in two individuals [48]. Considering that roe deer are now the most widespread species in the UK, it is possible that this lack of diversity may be due to a population bottleneck following the near-extinction of British roe deer prior to the 1800 s [37]. However, a similar lack of *PRNP* diversity was also evident in European roe deer populations from Italy [46], Scandinavia [48] and Spain [47]. Similarly, we found little evidence of sequence variation in sika, fallow and muntjac deer, with the result that each of these species is predicted to express a single specific variant of the PrP protein. However, with a relatively small number of animals analysed (103, 92 and 41 respectively), less frequent polymorphisms may still be found in larger surveys. The *PRNP* sequence we identified in British fallow deer (described here as TPE-N_138_), was identical to previously published sequences from Scandinavia [48] and Spain [47], as well as fallow deer previously used in experimental challenge experiments [34, 55]. Our data support the conclusion that the N_138_ change is fixed in the fallow deer sequence and not polymorphic. The three haplotypes identified in 27 wild Chinese water deer is more than might be expected considering that the UK population was established relatively recently from small numbers of escapees from captive collections.

Comparison of the *PRNP* genotypes in European deer with those in North American cervid species with European data can be used to help predict their potential susceptibility to CWD, although confirmation of these predictions under natural or experimental challenge is essential. The TPQ haplotype, which we identified at high frequencies in British red (0.29), Roe (1.0), Sika (1.0) and Chinese Water deer (0.21), is identical to *PRNP* sequences found in North American cervids that are highly susceptible to CWD. Our analysis, and published data to date for European deer species, has not identified any *PRNP* polymorphisms that are associated with reduced CWD susceptibility in North American cervids, e.g. M132L, G96S [25]. This suggests that a relatively high proportion of free-ranging/wild deer in Britain and Europe may be susceptible to CWD, or at least to the predominant strains of CWD in North America.

The association of non-synonymous *PRNP* polymorphisms found only in British/European deer with susceptibility to CWD has yet to be determined. Amino acid substitutions at codon 98 are found in red (T98A) and muntjac (T98S) deer, and nearby codons 95 and 96 play a role in CWD susceptibility in white-tailed deer [24]. However, muntjac deer with identical *PRNP* sequences can be experimentally infected with North American CWD isolates [31] and recently camels, which encode alanine at the equivalent position, were reported to develop natural prion disease [56].

The substitution of serine for proline at codon 168 in red deer is of particular interest, as this position is located in the β2-α2 loop of the prion protein, an area critical for protein structure and stability [57]. The murine prion protein with a serine substitution at the equivalent residue showed no difference to wildtype in efficiency of conversion to PrP^Sc^ in an in vitro cell-free conversion assay [57]. However, in sheep the substitution of leucine for proline at codon 168 was associated with very prolonged incubation periods for experimental BSE infection [58], and a reduction of cell-free conversion efficiencies [59]. The ASQ haplotype seen in red deer has not been tested in vitro or by prion challenge, so any potential association with prion disease is still unresolved.

The novel *PRNP* haplotype TPQ-L_247_ identified in Czech red deer may be less likely to influence susceptibility as it represents a conservative amino acid substitution within the C-terminal signal peptide which is cleaved from the mature prion protein [60], however it is possible that it may have unknown effects that influence pathogenesis.

In North America codon 226 encodes either E (e.g. wapiti) or Q (e.g. mule deer and white-tailed deer), whereas in European red deer both variants are present and heterozygous animals are common. Codon 226 is in the third α-helix of PrP^C^, a part of the protein involved in pathogenic processes during prion disease. Nearby polymorphisms include S225F, which is associated with reduced CWD susceptibility in mule deer [20], and Q222K, which is strongly associated with prion disease resistance in goats [61, 62]. Studies in transgenic mice expressing cervid PrP with Q226 or E226 suggest that variation at 226 may contribute to differences in disease progression and susceptibility to certain prion strains [63, 64]. Following experimental oral transmission of CWD to four deer of EE_226_, QQ_226_ and EQ _226_ genotypes, all animals became infected [65]. The small sample size of this study does not allow a definite conclusion regarding the association of codon 226 with CWD susceptibility, or the effect of heterozygosity at this position.

The TPQ-N_100_ haplotype identified in Chinese water deer has not been reported in any other cervid but N_100_ is common to other mammalian prion proteins including bank vole, a species highly susceptible to CWD challenges [66]. The fact that fallow deer failed to become infected with CWD when exposed to infected mule deer for up to 7 years suggests that the substitution of asparagine at codon 138 may be associated with relative resistance to infection, but it is possible that other species-specific genetic factors also play a role [34].

In summary, this study has provided an analysis of *PRNP* sequence variation in the six major deer species found in the wild in Great Britain and adds to our knowledge of *PRNP* variation in European deer species in general. Based on a comparison of the sequences with those found in North American cervids affected by CWD, it appears likely that a large proportion of the British deer population would be susceptible to CWD. The effect of specific *PRNP* haplotypes only found in European species, such as ASQ in Scottish red deer, on CWD susceptibility are still to be determined.

## Data Availability

The datasets used and/or analysed during the current study are available from the corresponding author on reasonable request.

## Abbreviations

CWD: chronic wasting disease
*PRNP*: prion protein gene
SNP: single nucleotide polymorphism
EU: European Union
GB: Great Britain
ORF: open reading frame
